# Retrospective cross-sectional observational study on the epidemiological profile of dengue cases in Pernambuco state, Brazil, between 2015 and 2017

**DOI:** 10.1186/s12889-020-09047-z

**Published:** 2020-06-12

**Authors:** Iasmyn Dayanne Santos do Nascimento, André Filipe Pastor, Thaísa Regina Rocha Lopes, Pablo Cantalice Santos Farias, Juliana Prado Gonçales, Rodrigo Feliciano do Carmo, Ricardo Durães-Carvalho, Caroline Simões da Silva, José Valter Joaquim Silva Júnior

**Affiliations:** 1 São Miguel University Center, Recife, Pernambuco Brazil; 2 Federal Institute of Education, Science and Technology of Sertão Pernambucano, Floresta, Pernambuco Brazil; 3 Virology Sector, Laboratory of Immunopathology Keizo Asami, Federal University of Pernambuco, Recife, Pernambuco Brazil; 4 Department of Virology, Aggeu Magalhães Institute, Oswaldo Cruz Foundation, Recife, Pernambuco Brazil; 5 Collegiate of Pharmaceutical Sciences, Federal University of Vale do São Francisco, Petrolina, Pernambuco Brazil; 6 Laboratory of Virology, University of Campinas, Campinas, São Paulo, Brazil; 7 Virology Sector, Department of Preventive Veterinary Medicine, Federal University of Santa Maria, Av. Roraima, Camobi, Santa Maria, Rio Grande do Sul 97105-900 Brazil; 8 Department of Microbiology and Parasitology, Federal University of Santa Maria, Santa Maria, Rio Grande do Sul Brazil

**Keywords:** Dengue, Epidemiology, Brazil, Northeastern Brazil, Pernambuco state

## Abstract

**Background:**

The spread of *Dengue virus* (DENV) infections, as well as their signs and symptoms, are the result of a complex interaction between several factors. In Brazil, especially in the Northeastern, dengue is an important public health problem. Here, we report an epidemiological analysis of dengue cases in Pernambuco state, Northeastern Brazil, during 2015–2017.

**Methods:**

This work is a retrospective cross-sectional observational study on the epidemiological profile of all dengue cases confirmed and reported to the Health Secretary of Pernambuco between 2015 and 2017. These data cover all municipalities of Pernambuco, except *Fernando de Noronha*. DENV-positive individuals were classified according to the dengue type (without and with warning signs, or severe dengue), age, gender, ethnicity and intermediate geographic region of residence (Recife, Caruaru, Serra Talhada or Petrolina). The distribution of cases over the years was assessed by χ2 test. Temperature and rainfall data were evaluated by Unpaired t-test. *p-value* < 0.05 and CI 95% were considered in all analyses.

**Results:**

Most dengue cases was without warning signs. The most observed characteristics in the less severe dengue phenotypes were: female, mulatto ethnicity and age between 20 and 39 years old; this profile was more clearly observed in 2015. In 2016 and 2017, however, the numbers of dengue without and with warning signs were more evenly distributed and the difference in cases within groups decreased significantly. Regarding severe dengue, mulattoes were the most affected, but it is possible to note a trend towards a more uniform distribution between the genders and ages. Recife was the region with the highest numbers of both total cases and incidence rates and the highest rainfall levels. Overall, over the years, there has been a decrease in dengue cases in all regions of Pernambuco.

**Conclusions:**

We identified the epidemiological profile of dengue in Pernambuco, Brazil, reporting the gender, age, ethnicity and regions most affected by different dengue types. In addition, we observed that these cases were probably more influenced by rainfall than by temperature. Finally, we believe that this epidemiological knowledge is important to direct public health policies to the reality of each population.

## Background

*Dengue virus* (DENV) is an arbovirus belonging to the family *Flaviviridae*, genus *Flavivirus*, and has four antigenically distinct serotypes, DENV-1, − 2, − 3 and − 4 [1]. Currently, the World Health Organization (WHO) estimates that 50 to 100 million new DENV infections occur worldwide each year and in more than 100 endemic countries [2]. It is believed that over 2.5 billion people, i.e., over 40% of the world’s population, are at risk of dengue fever, mainly in South and Central America, Africa and South Asia [2, 3].

The number of dengue cases is the result of a complex interaction between viral, environmental and host factors, which also influence the signs and symptoms of the disease. Regarding the host, herd immunity has historically influenced the incidence of dengue. The introduction of new serotypes into areas free of their circulation and/or the re-emergence of serotypes after considerable epidemiological silencing has been responsible for millions of infections [4–6]. In addition, the co-circulation of different DENV serotypes and the possibility of antibody-dependent enhancement (ADE) has been one of the main determinants of the severity of infections [7].

Several studies have also associated socio-environmental aspects with the number of dengue cases. Age, gender, ethnicity, education level or socio-economic status, for example, have already been related to the risk of DENV infections [8–15]. In relation to environmental factors, uncontrolled urbanization and climatic conditions, such as temperature and rainfall, capable of influencing the population of vector mosquitoes, mainly *Aedes aegypti* and *Ae. albopictus*, are some of the elements that contribute to the incidence of dengue [16–26]. Interestingly, the association between climatic factors and mosquito-borne infectious diseases, such as dengue and Zika, has also been demonstrated through information extracted from newspapers [27, 28].

In Brazil, a dengue-endemic country, outbreaks began to be frequent from the nineteenth century, initially in Rio de Janeiro state, Southeastern region [29, 30]. In recent years, the Northeastern region has also been highlighted as one of the regions with the highest number of cases and deaths by dengue. Between 2013 and 2018, for example, more than one million dengue probable cases and 707 deaths were reported, corresponding to 18.1 and 22.3% of all country, respectively [31–35]. During the same period, Pernambuco state was responsible for about 20.1% of the total probable cases and for 19.4% of deaths by dengue in the Northeast region [31–35].

Since 2015, the chikungunya and Zika viruses (CHIKV and ZIKV) have also circulated in Brazil, probably introduced in 2014 and 2015, respectively [36, 37]. The co-circulation of these three viruses, in turn, raised serious public health concerns and several studies have investigated its influence on vector transmission, ADE or cross-protection [38–43].

Although these investigations are important, we believe that knowledge of the epidemiological profile of dengue cases, especially in regions with arboviruses co-circulation, is also a useful tool for adapting public health policies. Therefore, we carried out an epidemiological characterization of dengue cases in Pernambuco, Brazil, in the 2015–2017 triennium, describing the gender, age, ethnicity and the regions most affected by the different dengue types (without and with warning signs, or severe dengue). In addition, we also collected temperature and rainfall data from Pernambuco and discussed their possible influences on dengue cases.

## Methods

### Study design

This work is a retrospective cross-sectional observational study on all dengue cases confirmed and reported to the Health Secretary of Pernambuco, Brazil, between the years 2015 and 2017.

### Region and population of study

**Fig. 1:**
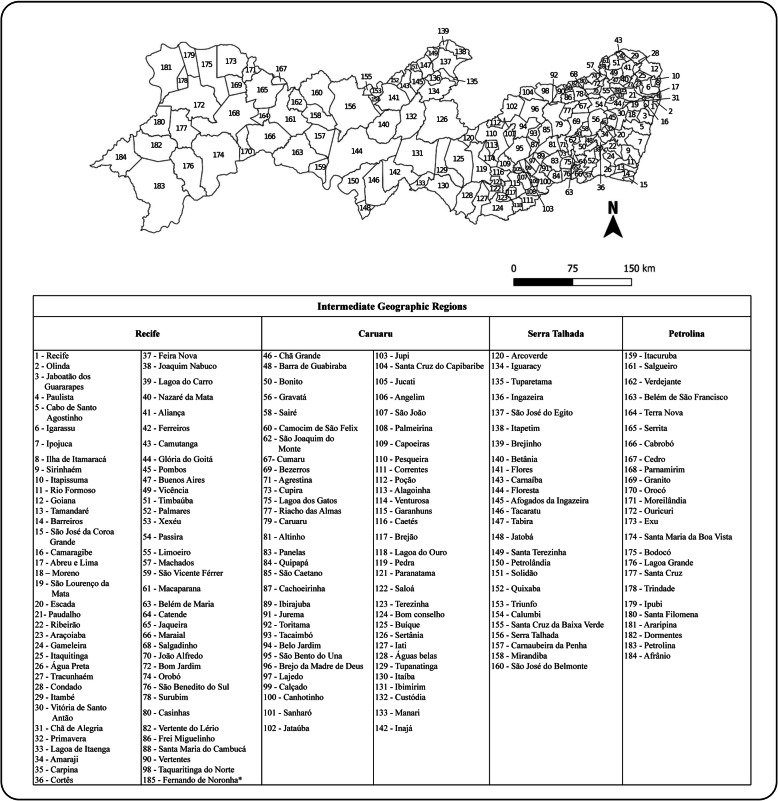
Municipalities and intermediate geographic regions of Pernambuco state, Brazil. The distribution of the municipalities in each intermediate region is in accordance with data from the Brazilian Institute of Geography and Statistics (IBGE). *Fernando de Noronha* municipality (island) is not shown in the map. Map made available by *Suporte Geográfico* (free access, without copyright) (https://suportegeografico77.blogspot.com/2018/04/mapa-municipios-de-pernambuco.html?m=1) and adapted in WPS Office 2016 Free (version: 11.2.0.93.63)

The Pernambuco state is located in the northeast of Brazil and occupies an area of 98,312km^2^. Currently, Pernambuco is divided into four intermediate geographic regions: Recife, Caruaru, Serra Talhada and Petrolina, which have 72, 63, 25 and 25 municipalities, respectively [44] (Fig. 1). In this study, the total population of each region corresponded to the sum of the estimated population of its municipalities, according to data available at the Brazilian Institute of Geography and Statistics (*Instituto Brasileiro de Geografia e Estatística*, IBGE - http://www.ibge.gov.br).

### Data collection

#### Dengue cases

All dengue cases confirmed and reported to the Health Secretary of Pernambuco between 2015 and 2017 were used in the study. The cases cover all municipalities of Pernambuco, except *Fernando de Noronha*. For each dengue-positive individual, information on clinical classification (dengue without and with warning signs, or severe dengue), gender, ethnicity, age and intermediate region of residence were collected. Clinical classification was performed according to the WHO, using clinical and/or laboratory criteria [45]. The number of cases in each intermediate region corresponded to the sum of the data from all the municipalities that compose it. All this information were made available by the Health Secretary of Pernambuco after formal request (protocol number 0026390–2/2018).

#### Temperature and rainfall

We also analyzed rainfall and temperature data from the intermediate geographic regions of Pernambuco. These data were obtained from the Pernambuco State Agency for Water and Climate (*Agência Pernambucana de Águas e Clima*, APAC) database (http://www.apac.pe.gov.br). The annual temperature was obtained from the thermal scale analysis of the Pernambuco map, followed by the arithmetic mean of the minimum and maximum temperatures in the 12 months of the year. Rainfall data were obtained from monthly rainfall bulletins of the municipalities of Pernambuco. *Fernando de Noronha* municipality was excluded from the climatic analysis.

### Statistical analyses

Statistical analyses were performed using GraphPad Prism software v.6.07. Temperature and rainfall data from the intermediate regions were evaluated by Unpaired t-test. The distribution of dengue cases during 2015–2017 was assessed by χ2 test. The *p-value* < 0.05 and CI 95% were considered in all analyses.

## Results

**Fig. 2:**
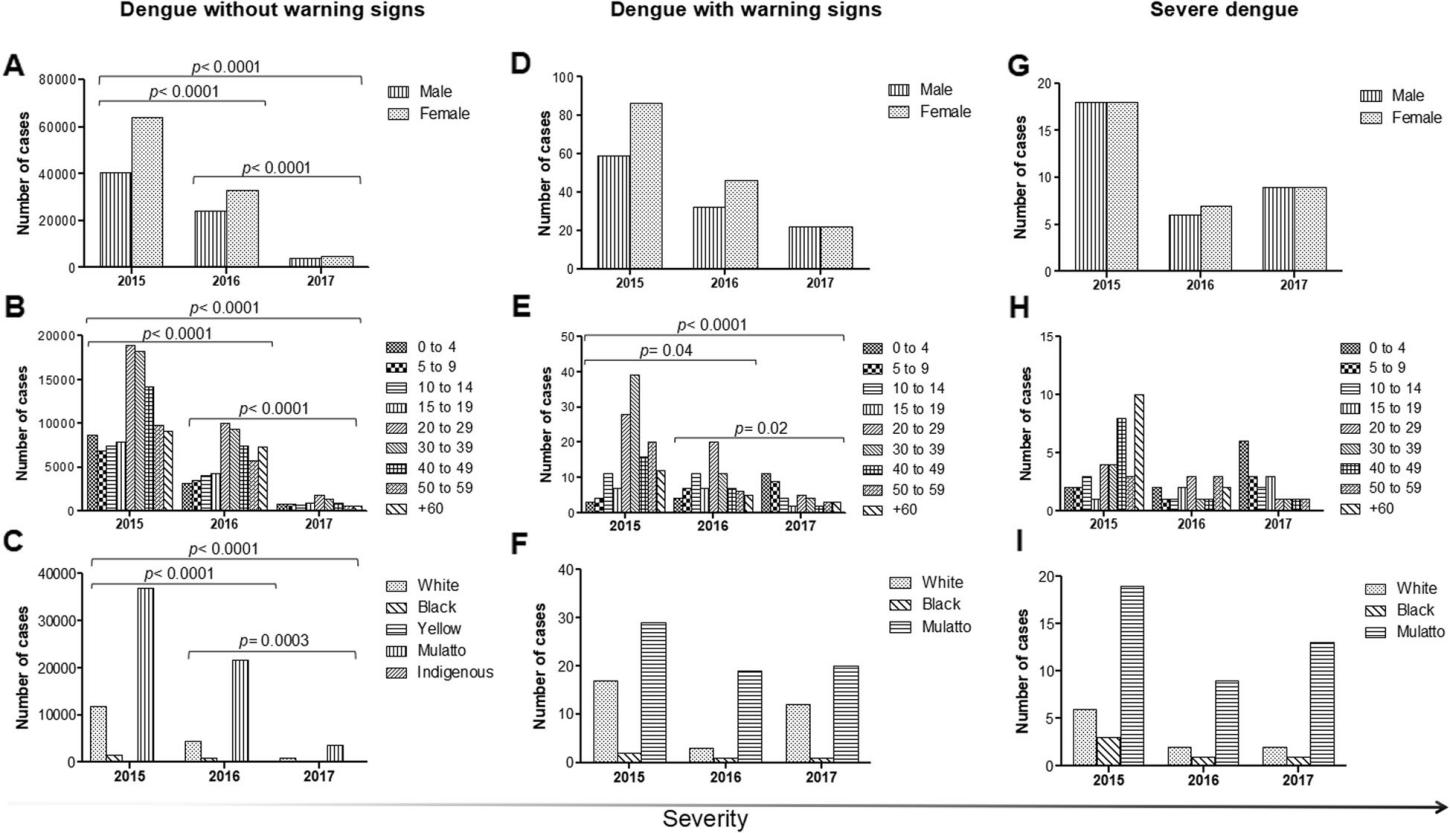
Number of dengue cases without and with warning signs and severe dengue according to gender, age group and ethnicity. The analyses were performed with data collected between 2015 and 2017 from all municipalities of Pernambuco, except *Fernando de Noronha*. During case reporting, among the 170,477 individuals diagnosed (total cases), ethnicity was ignored in 87,496. **a**, **b** and **c**: correspond to the number of dengue without warning signs cases, according to gender, age and ethnicity of infected individuals, respectively; **d**, **e** and **f**: number of dengue with warning signs cases according to gender, age and ethnicity of infected individuals, respectively; and **g**, **h** and **i**: number of severe dengue cases according to gender, age and ethnicity of infected individuals, respectively. The *p-value* was obtained by χ2 test

Over the 3 years, we observed that the majority of dengue cases was without warning signs. The highest number of cases without warning signs occurred in female (Fig. 2a), individuals aged 20–29 and 30–39 years old (Fig. 2b), and mulattoes (Fig. 2c). This profile was also observed for dengue with warning signs (Fig. 2d-f) and for the total number of dengue, i.e., without distinction between clinical manifestation (data not shown).

The epidemiological profile described above was clearly observed in 2015. In the following years, 2016 and 2017, there was a more uniform distribution of the less severe dengue phenotypes in relation to at least one of the characteristics evaluated. For dengue without warning signs, this change can be verified by the significant decrease in the difference in cases between the genders (2015 vs. 2016, 2016 vs. 2017 and 2015 vs. 2017, *p* < 0.0001) (Fig. 2a), age groups (2015 vs. 2016, 2016 vs. 2017 and 2015 vs. 2017, *p* < 0.0001) (Fig. 2b) and ethnicities (2015 vs. 2016 and 2015 vs. 2017, *p* < 0.0001; 2016 vs. 2017, *p* = 0.0003) (Fig. 2c). Regarding dengue with warning signs, although it is possible to observe a trend towards homogenization in relation to population characteristics, the differences between the number of cases were significantly reduced only in the age groups (2015 vs. 2016, *p* = 0.04; 2016 vs. 2017, *p* = 0.01; and 2015 vs. 2017, *p* < 0.0001) (Fig. 2e).

With regard to severe dengue, the highest number of cases has also reported in mulatto individuals (Fig. 2i). However, the severe disease was more distributed between the genders (Fig. 2g) and its incidence varied widely in relation to the different age groups during the 3 years of analysis (Fig. 2h).

**Fig. 3:**
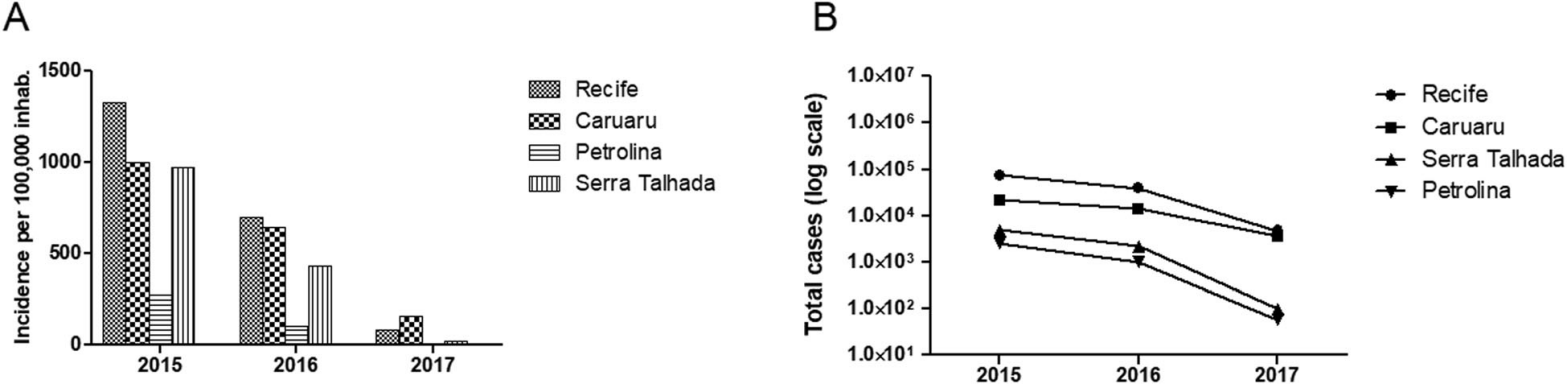
Number of dengue cases in the intermediate geographic regions of Pernambuco. **a**: incidence per 100,000 inhabitants in each intermediate region of Pernambuco. The values were based on the total number of individuals from each region available at the Brazilian Institute of Geography and Statistics (IBGE). **b**: total numbers of dengue cases in the intermediate regions of Pernambuco. In both analyses, all dengue cases reported to the Health Secretary of Pernambuco between 2015 and 2017 were used. These data cover all municipalities of Pernambuco, except *Fernando de Noronha*

Between 2015 and 2017, the intermediate regions of Recife and Caruaru had the highest numbers of cases per 100,000 inhabitants (Fig. 3a), as well as the highest numbers of total cases (Fig. 3b). In addition, both incidence per 100,000 inhabitants (Fig. 3a) and total numbers of cases (Fig. 3b) decreased in all intermediate geographic regions during the 3 years (Fig. 3a-b).

**Fig. 4:**
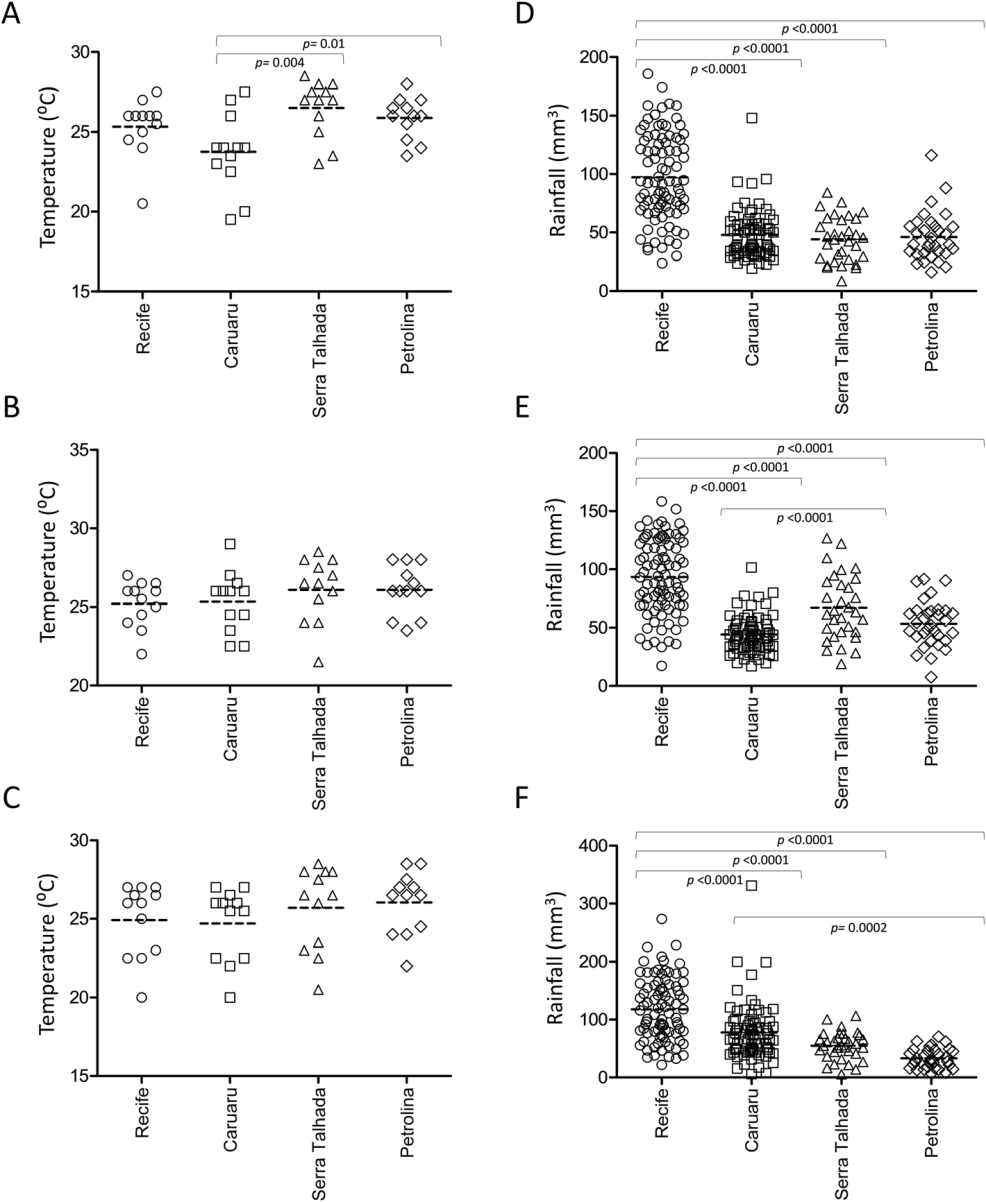
Temperature and rainfall means in the intermediate geographic regions of Pernambuco state, Brazil, between 2015 and 2017. **a**, **b** and **c**: the symbols correspond to the mean temperature for the months of 2015, 2016 and 2017, respectively; **d**, **e** and **f**: the symbols represent the mean monthly rainfall for each municipality of Pernambuco in 2015, 2016 and 2017, respectively. *Fernando de Noronha* municipality was excluded of the climatic analyses. Temperature and rainfall data for each intermediate region were obtained from the Pernambuco State Agency for Water and Climate (APAC). The *p-value* was obtained by Unpaired t-test

**Fig. 5:**
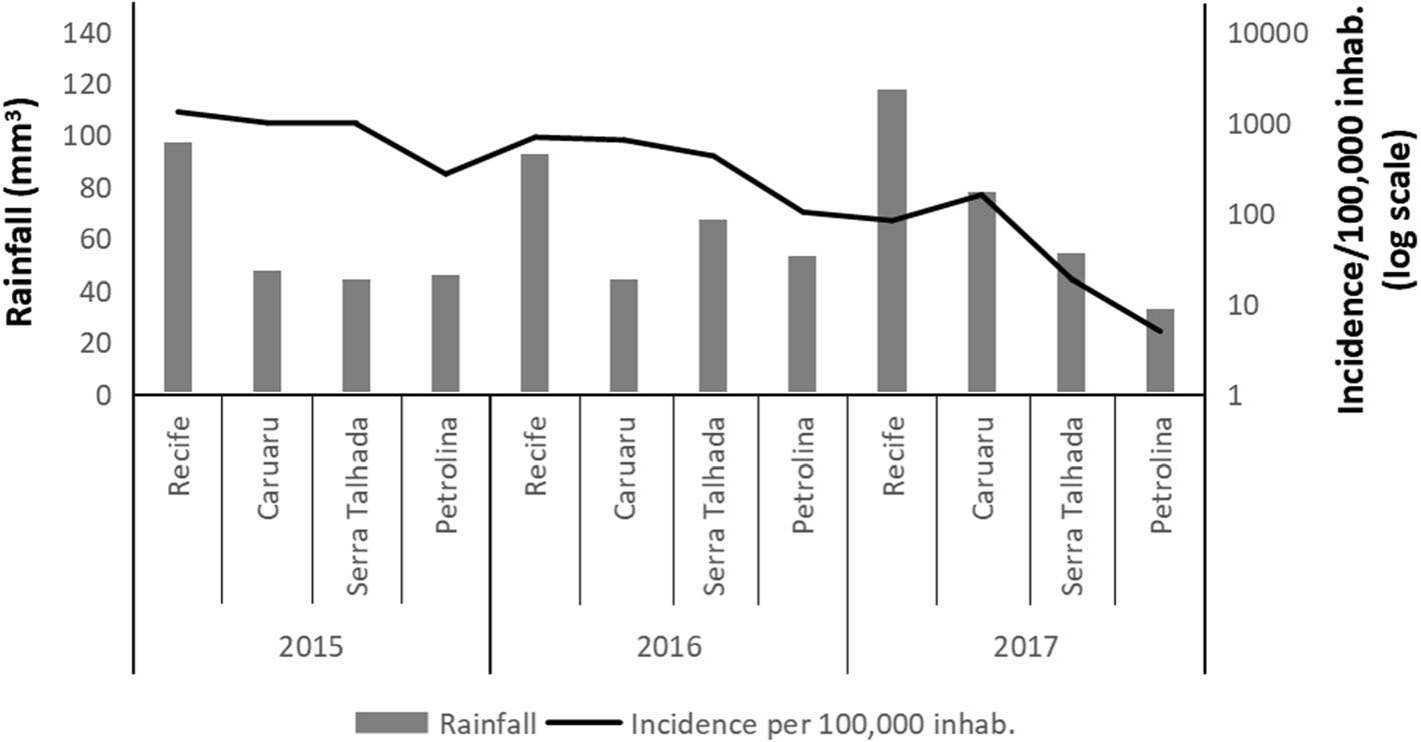
Dengue cases vs. Rainfall levels. Y axis on the left: mean rainfall for each intermediate region. Data obtained from the Pernambuco State Agency for Water and Climate (APAC). Y axis on the right: dengue cases per 100,000 inhabitants (expressed in base-10 logarithmic scale). Values were based on the total number of individuals from each region available at the Brazilian Institute of Geography and Statistics (IBGE) and in all dengue cases reported to the Health Secretary of Pernambuco between 2015 and 2017

Climate analyses revealed that the annual temperatures of the four intermediate regions did not differ significantly over the 3 years (Fig. 4a-c), except in Caruaru, which was colder than Serra Talhada and Petrolina in 2015 (Fig. 4a). On the other hand, the intermediate region of Recife presented the highest rainfall means in the 3 years (Fig. 4d-f). Finally, when the numbers of cases per 100,000 inhabitants and the mean rainfall were plotted together, it is possible to observe the increase in cases in some regions with the highest rainfall means (Fig. 5).

## Discussion

The incidence and clinical manifestations of dengue are strongly influenced by viral, environmental and host factors. With regard to host, in addition to the elements closely related to immunity and genetic background, socio-demographic characteristics have also been associated with the number and severity of DENV infections [11, 17, 23, 46–48]. In this perspective, we report the epidemiological profile of dengue cases in Pernambuco state, Brazil, in the 2015–2017 triennium.

Among the various social factors, age is one of the characteristics most related to DENV infections [9, 11, 14, 49–51]. In Pernambuco, during the 3 years studied, we observed that the majority of dengue cases was reported in individuals aged between 20 and 29 and 30–39 years old. A previous study carried out in Brazil between 2014 and 2016, in Rio Grande do Sul state, Southern region, also found a higher incidence of dengue cases in individuals aged 21–40 years old [52]. The similarity between these findings is interesting, especially considering that the studies were conducted in different years and in regions located at the extremes of Brazil, which have considerable climatic, cultural and genetic differences [53].

Analysis conducted with Korean travelers found a relationship of individuals aged 20–29 years old and the risk of DENV infection [10]. The study of Yung et al. [11] related the age group of 21–40 years old with highest seropositivity for DENV; interestingly, this group was equivalent to the most prevalent age groups in our study, i.e., 20–29 and 30–39 years old. In different regions and in different population segments, other age groups, e.g. infants and individuals with 15–49 years, 18 years, ≥ 30 years or > 50 years, have been also related to DENV infection [9, 11, 14, 50, 51].

In our study, female was the most affected by DENV infections throughout the analyzed period. This finding have not been observed in other studies. In Rio Grande do Sul state, the number of dengue cases in males was slightly higher than those reported in females in the years 2014 and 2016 [52]. A cross-sectional survey conducted in Brazil, but in the North region, precisely in rural Amazonia, observed a relationship between male and seropositivity for DENV [8]. Similarly, Korean male travelers had an increased risk of DENV infection [10]. In Lahore, Pakistan, a cross-sectional descriptive study found that the majority of individuals infected with DENV was male [54].

Regarding ethnicity, mulattoes represented the majority of the individuals diagnosed with dengue in Pernambuco between 2015 and 2017. Different ethnicities have been related to the risk of DENV infection. In Brazil, another study performed in Pernambuco identified that children born to mothers of Caucasian/Asian descent had a high risk of dengue [12]. In Singapore, Malay ethnicity was related to protection against DENV infections in a study with patients diagnosed between 2005 and 2013 [11]. In Colombia, it was reported that the Afro-Colombian population had a lower risk of DENV infection than non-Afro-Colombians [55].

When our data were analyzed according to the different clinical manifestations of dengue, the epidemiological profile was similar to the characteristics found for the total dengue cases. Indeed, it is possible to observe a higher incidence of dengue in mulatto individuals, female and aged between 20 and 29 and 30–39 years old, mainly in 2015 and in the less severe forms of dengue. However, over the 3 years, we noted a tendency towards an equal incidence of dengue without warning signs cases in relation to genders, age groups and ethnicities and of dengue with warning signs cases in relation to ages.

Regarding severe dengue, we observed a higher prevalence in mulattoes and a uniform distribution between the genders. Although this finding may be influenced by the low number of diagnosed individuals, this report is interesting since female is the gender that has been more often associated with the most severe cases of dengue [46, 49, 56]. As reviewed by Whitehorn and Simmons [46], it is possible that behavioral factors and/or physiological and immunological differences influence the pathogenesis of dengue in women.

In 2017, severe dengue was more distributed in the young population, especially in children. The involvement of the most severe forms of dengue in this population has been reported in other studies [46, 50, 51, 57]. Briefly, in newborns, maternal anti-DENV antibodies transmitted to fetuses can protect them from DENV infections. However, after the fourth or sixth month of life, the ADE outweighs neutralization and the infant has an increased risk to develop dengue severe forms [49, 51]. Furthermore, it is believe that the highest vascular endothelial permeability of children may also influence the clinical complications of dengue [57].

Among the intermediate regions of Pernambuco, Recife and Caruaru had the highest number of cases and the highest dengue incidence per 100 thousand inhabitants; a scenario possibly influenced by environmental factors, mainly climatic conditions, which strongly modulate the population of vector mosquitoes [15, 17, 19–26]. The Pernambuco state, however, located in the tropical region of Brazil, precisely between the Equator and the Tropic of Cancer, is marked by constant temperatures, as evidenced by our temperature analysis of the four intermediate regions over the 3 years. In this context, although detailed epidemiology analyses are required, we do not believe that the number of dengue cases has been directly related to the temperature during the analyzed period.

On the other hand, regarding rainfall, we observe that Recife was the region with the highest rainfall means during the 3 years and the region with the most dengue cases (both total cases and incidence per 100 thousand inhabitants) in 2015 and 2016. In addition, it is also possible to observe a significant difference of rainfall between the regions with more and less dengue cases over the 3 years, mainly between Recife and Petrolina, respectively. In this context, although the incidence of dengue is influenced by different factors, we believe that in Pernambuco, during 2015–2017, rainfall was probably one of the main factors responsible for the difference in the incidence of dengue among the intermediate regions.

In agreement with our findings, in Paraíba state, also in the Northeast region of Brazil, a significant association was observed between rainfall and dengue cases whereas temperature was not found to be a useful predictor [58]. The influence of rainfall on the density of *Ae*. *aegypti* and *Ae*. *albopictus* mosquitoes and the increase of dengue cases has also been reported in other Brazilian regions, such as in the North region [23, 59], as well as in other continents [16, 19, 21, 23, 25, 60].

In a chronological analysis performed during 2015–2017, it was possible to observe a decrease of dengue cases in all regions of Pernambuco. Interestingly, this decrease is observed throughout the Brazilian Northeast and in the general numbers of the country [32–34]. Despite the several factors capable of influencing the number and severity of DENV infections, such as environmental conditions, vector control measures, population immunity and co-circulation with CHIKV and ZIKV, the cause of this reduction in Brazil has not yet been fully clarified [61]. With reference to Pernambuco, we encourage future multidisciplinary studies, combining mainly virology, entomology and environmental science, so that the reduction of dengue cases in the state can be better understood.

One of the limitations of the study was that we did not have access to information about the economic status and educational level of individuals diagnosed with dengue. In addition, the ethnicity of most positive individuals was ignored during notification of infection. We believe that these data are important and would strengthen our analyses. Nevertheless, the epidemiological profile outlined here is a considerable basis for understanding the epidemiology of dengue in Northeastern Brazil, and we encourage further socio-environmental studies in areas not yet epidemiologically characterized, especially in dengue-endemic regions.

## Conclusions

Overall, between 2015 and 2017, the majority of dengue cases in Pernambuco state, Brazil, was diagnosed in 2015, in mulattoes, females, individuals with 20–39 years old and in the intermediate geographic regions of Recife and Caruaru. This epidemiological profile was most clearly observed in dengue without and with warning signs and in the first years of analysis. Interestingly, over the years and in severe dengue cases, there was a tendency towards a uniform distribution of cases in relation to the population characteristics. Regarding the climatic aspects, we believe that during the 3 years of analysis, the rainfall influenced the number of dengue cases more than the temperature.

Finally, although there are specific individual and social characteristics capable of influencing dengue infection and/or its severity, it is important to highlight that there is no universal epidemiological profile of the target population. Therefore, it is necessary that each region, especially the endemic areas, knows in detail the characteristics of its population and periodically update this information, so that public health policies can be directed to each social reality.

## Data Availability

Regarding dengue cases in the municipalities from Pernambuco state, Brazil, the data that support the findings of this study are available from the Health Secretary of Pernambuco, but restrictions apply to the availability of these data, which were used under license for the current study, and so are not publicly available. Data are however available from the authors upon reasonable request and with permission of the Health Secretary of Pernambuco. Data on dengue case in Brazil and its Northeast region are available on the Brazilian Ministry of Health repository, http://www.saude.gov.br/boletins-epidemiologicos. Data of temperature and rainfall were obtained from the APAC database, http://www.apac.pe.gov.br.

## Abbreviations

ADE: Antibody-dependent enhancement
APAC: Pernambuco State Agency for Water and Climate (*Agência Pernambucana de Águas e Clima*)
CHIKV: *Chikungunya virus*
CI: Confidence interval
DENV: *Dengue virus*
IBGE: Brazilian Institute of Geography and Statistics (*Instituto Brasileiro de Geografia e Estatística*)
WHO: World Health Organization
ZIKV: *Zika virus*
